# Thyroid Diseases Are an Underestimated Risk Factor for Cerebral Venous Sinus Thrombosis

**DOI:** 10.3389/fneur.2020.561656

**Published:** 2020-10-22

**Authors:** Maren Hieber, Charlotte von Kageneck, Cornelius Weiller, Johann Lambeck

**Affiliations:** Clinic of Neurology and Neurophysiology, Medical Center–University of Freiburg, Faculty of Medicine, University of Freiburg, Freiburg, Germany

**Keywords:** cerebral venous sinus thrombosis, risk factors, thyroid disease, hyperthyroidism, hypercoagulability

## Abstract

**Introduction:** Cerebral venous sinus thrombosis (CVST) is a rare disease that generally accounts for just 1% of all strokes. Of the multiple risk factors that have been identified, the most common are genetic or acquired thrombophilia and the use of oral contraceptives, while the less common include local infections and mechanical causes. Thyroid diseases have been described as rare risk factors for CVST (<2% of all cases), without exact knowledge of the underlying pathophysiology. This retrospective study aimed to re-evaluate the relevance of thyroid disease as risk factor for CVST, with particular emphasis on hyperthyroidism.

**Patients and Methods:** Confirmed cases of CVST were (re-)evaluated in terms of risk factors including thyroid parameters. Results were compared to previous data from the International Study on CVST.

**Results:** Between 1996 and 2016, 182 patients with confirmed CVST were treated in our hospital with a median age of 44 years and a female proportion of 74.7%. Genetic or acquired thrombophilia along with the use of oral contraceptives were found to be the most common risk factors. Thyroid diseases were present in 20.9% of CVST patients; this included patients with previous (9.9%) and current thyroid dysfunction (11%).

**Discussion and Conclusions:** Thyroid diseases may represent a more common risk factor for CVST than previously described. This holds true even if patients with current thyroid dysfunction are purely taken into account. However, 58% of patients had more than one additional risk factor, suggesting a multifactorial hypercoagulability.

**Clinical Trials Register**: Registered at the German Clinical Trials Register: http://www.drks.de, DRKS00017044.

## Introduction

Cerebral venous sinus thrombosis is a rare disease with an estimated incidence of around 0.5/100.000/year (1, 2) to 2/100.000/year (3, 4), and only accounts for a small proportion (1%) of all strokes. The possible underlying causes or risk factors span a broad spectrum of conditions. These range from focal-like infections of the central nervous system, as well as traumas or arteriovenous malformations to systemic disorders such as cancer, hematological disorders or prothrombotic conditions, both genetic and acquired. Along with oral contraceptives, the latter represent the most common risk factors. Whereas, more than one risk factor is detectable in many patients with CVST, a relevant proportion of 12.5% of cases remains in which no risk factor is identified at all (5).

Several case reports, small clinical trials and basic research studies generally suggest that thyroid diseases, especially hyperthyroidism, serve as a risk factor for CVST or thromboembolism. Existing data indicate a possible association between hyperthyroidism and moderate hypothyroidism with a state of hypercoagulability (6–9). Furthermore, several case reports describe patients with CVST and the additional condition of hyperthyroidism [e.g., (10–12)]. In the majority of reported cases, hyperthyroidism was due to Graves' disease. In contrast, Pekdemir et al. stated that chronic thyroiditis without further differentiation was the reason for the hyperthyreote state in their patient (11). Mouton et al. reported one case of postpartum thyroiditis in one of their four patient cases (10). The International Study on CVST (ISCVT) includes thyroid diseases amongst a large number of other various risk factors, albeit with a very low fraction (1.7%) (5). While this occurrence rate remains below the common prevalence of thyroid dysfunction in Europe (3.8%) (13), Verberne et al. report a significantly higher incidence of combined CVST and thyrotoxicosis than expected by chance alone (14). In contrast to this association of hyperthyroidism (and moderate hypothyroidism) and hypercoagulability, overt hypothyroidism seems to be associated with an increased bleeding tendency (15–17).

Following a case of Grave's disease with CVST in our department (12), we wanted to re-examine various known risk factors for CVST and determine their numerical relevance, with particular emphasis on the role of thyroid disease.

## Patients and Methods

We performed a retrospective single center analysis of all documented cases of CVST in our department, and re-evaluated the risk factors and causes, including thyroid diseases. In order to find all of these documented cases, we performed a computerized full-text search for the term “CVST” in all text documents that were electronically saved in the local documentation system between 1996 and 2016. This included patient letters for those who were not primarily treated at the stroke unit or neuro-ICU in our department (providing the concomitant condition was more severe and dominant), but were rather seen by one of our consulting specialists. The search revealed a list of patients whose electronic documentation was then screened manually by two neurologists to identify all confirmed diagnoses of CVST (including cases of isolated cortical vein thrombosis). In all confirmed cases, the complete electronic documentation was examined for any risk factors recorded at the time of diagnosis, as well as possible other risk factors (insofar as they could be evaluated within the documentation). The resulting database was used for quantitative analysis, which was additionally compared to the results of the International Study on CVST (ISCVT). The presence of thyroid disease was generally determined in two ways: (i) the patient had a positive history of thyroid disease (based on either the patient's statement or previous reports and findings), which was then referred to as a “previous thyroid disorder,” or (ii) laboratory findings at the time of CVST diagnosis repeatedly revealed pathological thyroid parameters (referred to as “current thyroid disease”). The latter was then further differentiated into: (i) current hypothyroidism (defined by elevated levels (> 4.2 mU/l) of thyroid-stimulating hormone, TSH) with or without reduced levels of free T_4_ (= manifest and subclinical hypothyroidism, respectively) (18), and (ii) current hyperthyroidism (defined by reduced levels (<0.27 mU/l) of TSH) with or without elevated levels of T_4_ and T_3_. (= manifest and subclinical hyperthyroidism, respectively) (19). Analysis of thyroid-specific auto-antibodies was additionally performed, especially in cases of current manifest hyperthyroidism.

The study was approved by our local ethics committee (Ethics Committee of the University of Freiburg, Germany; IRB number 229/19; clinical trial registration DRKS00017044). Due to the retrospective format of the study, informed consent was not obtained from the included patients.

## Results

**Figure 1:**
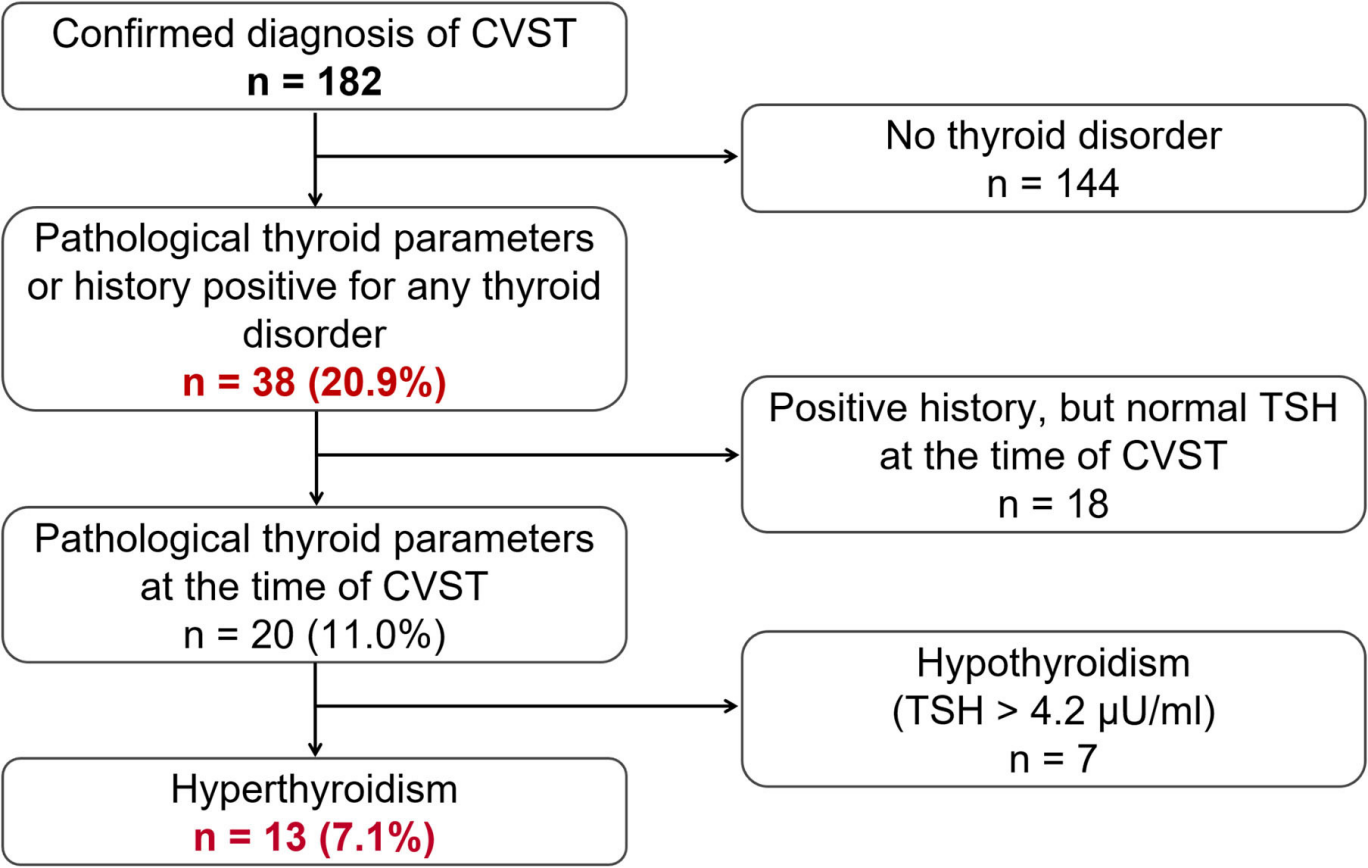
Flowchart showing the general frequency of thyroid disease (*n* = 38, 20.9%) as a risk factor for CVST, and the respective subgroups of actual or previous pathological thyroid parameters and actual hyper- or hypothyroidism.

The computerized full-text search yielded 1,303 patient datasets, in which the term “CVST” was present in the text documents. Further manual screening led to the exclusion of 1,121 cases, due to the lack of a final diagnosis for CVST. In these particular documents, the term “CVST” mainly appeared as “exclusion of CVST” or “no hint of CVST” in the neuroradiological reports. We identified 182 cases that each had a confirmed diagnosis of CVST that was validated by CT- or MR-venography. All electronically available documents, reports and findings related to these 182 patients were (re-) evaluated for baseline characteristics (Table 1), medical treatment and risk factors (see Table 2), including thyroid diseases. By analyzing the annual number of CVST patients treated in the 20-year study period, an average of nine CVST patients were treated per year in our department. Table 3 lists the number of CVST patients per year. Leaving aside small fluctuations in the incidence of CVST at some timepoints, the number of patients diagnosed with CVST continuously increased each year since 1996. Since 2008, more than ten patients per year were diagnosed with CVST and treated accordingly, a finding consistent with the results of another German single-center study (20). Baseline characteristics of the 182 CVST patients revealed a median age of 44 years (range: 18–88 years). Approximately three quarters of the patients were female (74.7%). The affected sinuses and veins were (in descending order) the left lateral sinus (*n* = 94), the right lateral sinus (*n* = 78), the superior sagittal sinus (*n* = 74), the jugular veins (*n* = 39), the straight sinus (*n* = 18), cortical veins (*n* = 10), and deep veins (*n* = 6). (Re-)evaluation of risk factors yielded 45 patients (24.7%) without identifiable risk factors, while 86 patients (47.3%) exhibited more than one risk factor. The most common risk factor was thrombophilia, which was found in 53 patients (29.1%). This could be further distinguished into genetic thrombophilia (*n* = 33, 18.1%), mainly due to heterozygous mutation of factor 5 or factor 2, and probable acquired thrombophilia (*n* = 30, 16.5%), shown by antiphospholipid syndrome, hyperhomocysteinemia and an elevation of lupus anticoagulant. The second leading risk factor was the intake of oral contraceptives (*n* = 36, 19.8%), which also represented the most common risk factor in the subgroup of females under 50 years of age. A current or past history of thyroid dysfunction was detected in 38 patients (20.9%), which resulted in general thyroid dysfunction being the third leading risk factor for CVST. Eighteen (9.9%) of these CVST patients had a positive history for thyroid disease and/or were currently on medication (i.e. mostly L-thyroxine), but had normal thyroid lab parameters at the time of CVST (referred to as “former thyroid disease” in Table 2). Pathological thyroid parameters were present in 20 patients (11.0%) at the time of CVST (referred to as “current thyroid disease” in Table 2). This could be further distinguished into 7 patients with hypothyroidism and 13 patients with hyperthyroidism (7.1%) (see flowchart in Figure 1). In 16 of the 38 patients with a current or past history of thyroid dysfunction, no further risk factors were found. Of the 38 patients with any kind of thyroid disorder, 22 had at least one additional risk factor. Five out of the 13 patients with current hyperthyroidism showed no further risk factors. The additional risk factors in the group of current hyperthyroidism patients who had at least one further risk factor (*n* = 8) were genetic thrombophilia (*n* = 1), acquired thrombophilia (*n* = 1), systemic inflammatory disease (*n* = 1), puerperium (*n* = 1), infection (*n* = 1), mechanical precipitants (*n* = 1), oral contraception (*n* = 1), and other drugs (*n* = 2). No additional risk factors were found in one of the seven patients with current hypothyroidism. Further risk factors in the remaining six patients included genetic thrombophilia (*n* = 1), malignancies (*n* = 2), pregnancy (*n* = 1), infection (*n* = 2), and oral contraception (*n* = 1).

**Table 1:** Baseline and clinical characteristics of study cohort.

**Baseline and clinical characteristics**
Median age [years (range)]	44 (18, 88)
Female [*n* (%)]	136 (74.7)
**Affected sinus (>1 possible)**	
Left lateral sinus [*n* (%)]	94 (51.7)
Right lateral sinus [*n* (%)]	78 (42.7)
Superior sagittal sinus [*n* (%)]	74 (40.7)
Jugular vein [*n* (%)]	39 (21.4)
Straight sinus [*n* (%)]	16 (8.8)
Cortical veins [*n* (%)]	10 (5.5)
Deep veins [*n* (%)]	6 (3.3)

*Displayed are the median age (and range), and the absolute numbers [n] and the percentage [%] respectively*.

**Table 2:** Frequencies of the various risk factors in our cohort (left) in compared to those previously reported by the International Study on CVST (ISCVT).

**Risk factor**			**Our cohort [%]**			**ISCVT [%]**		
**Thrombophilia**	Genetic		**29.1**	18.1		**34.1**	22.4	
	Acquired	APS		16.5	13.7		15.7	5.9
		Homocysteine ↑			1.7			4.5
		Lupus anticoagulant ↑			3.3			n.a.
Oral contraceptives^*^			**40.0**			**54.3**		
**Thyroid disorders**	Current	Hyper-/Hypothyroidism	**20.9**	11.0	7.1/3.9	**1.7**	n.a.	n.a.
	Previous	Hyper-/Hypothyroidism		9.9	1.7/8.2		n.a.	n.a.
**Infection**			**10.4**			**12.3**		
**Malignancy**	Solid (not CNS)		**7.1**	5.5		**7.4**	3.2	
	Hematological			1.7			2.9	
Pregnancy^*^			**6.7**			**6.3**		
**Hematological condition**	Anemia^**^		**6.1**	5.0		**12.0**	9.2	
	Polycythemia			1.1			2.8	
**Drugs (w/o oral contraceptives)**	Steroids		**5.5**	4.4		**7.5**	1.6	
	Hormone replacement			1.1			4.3	
**Systemic inflammatory disease**			**3.9**			**1.8**		
**Mechanical precipitants (lumbar puncture, cranial trauma, jugular catheter occlusion and neurosurgery)**			**1.7**			**4.5**		
**CNS disorders (e.g., AV fistula)**			**1.7**			**1.9**		
**Dehydration**			**1.7**			**1.9**		
Puerperium^*^			**1.1**			**13.8**		
**Vasculitis**			**1.1**			**3.0**		

*APS Antiphospholipid antibody syndrome*.

↑*increase*.

* *fraction of females under 50 years*.

** *anemia was defined as Hb under 9 g/dl in our cohort, the definition used in ISCVT not available*.

**Table 3:** Number of CVST patients diagnosed and treated in our department between 1996 and 2016.

**Year**	**Number of CVST cases**	**Year**	**Number of CVST cases**
1996	1	2007	8
1997	0	2008	13
1998	2	2009	11
1999	3	2010	15
2000	0	2011	15
2001	0	2012	13
2002	4	2013	17
2003	3	2014	15
2004	5	2015	15
2005	12	2016	16
2006	14		

Of the 38 patients with a former or current thyroid disorder, three had Hashimoto's thyroiditis and two had Grave's disease. None of the patients had a known thyroid malignancy.

Only two patients were treated for recurrent CVST in our department; neither of these had any kind of thyroid disorder, nor were they treated in our clinic for the first occurrence CVST. One patient showed no risk factors, whilst the other was diagnosed with a hitherto undetected antiphospholipid antibody syndrome.

## Discussion

Our retrospective single-center analysis of all documented cases of CVST (1996–2016) revealed thyroid diseases to be the third most relevant risk factor for CVST, following the two leading risk factors (genetic or acquired) of thrombophilia and oral contraceptives. While the latter two factors and the patient baseline characteristics are in line with the results of the ISCVT (see Table 1 for a comparison), the frequency of thyroid disorders in our CVST cohort (20.9%) exceeds both the proportion of thyroid disorders reported in the ISCVT (1.7%) (5) and the common prevalence of thyroid disorders in Europe (3.8%) (13). The exact definition of “thyroid disorder” used in the study protocol of ISCVT is unclear. Our analysis distinguished between patients with either current or previous thyroid disorders as well as both hypo- and hyperthyroid disorders. Even when cases of current hyperthyroidism at the time of CVST were purely taken into account–an association which is supported by the majority of case reports and trials—our cohort proportion of 7.1% still exceeded that stated in the ISCVT (1.7%). This is in line with previous results stating that the incidence of combined CVST and thyrotoxicosis is significantly higher than that expected by chance alone (14).

One reason for the different proportions of thyroid disorders in our cohort vs. that of the ISCVT could be an epidemiological one characterized by a geographically-based iodine deficiency, particularly in southern Germany (21). Indeed, iodine insufficiency (22) has ameliorated by iodised salt prophylaxis, which has led to the sufficient iodine status in Germany at present (23); however, since our study dates back to 1996, there still may be a higher prevalence of iodine deficiency in our cohort, which is a known risk factor for hyperthyroidism (24, 25).

Another reason could be related to methodology: in terms of the lack of clarity regarding the exact definition of “thyroid disorders” in the ISCVT, one could argue that we may have used a broader definition, resulting in an increased prevalence. To minimize this risk, we have presented data from the complete spectrum of disorders, ranging from 20.9% for the broadest definition (including all former and current thyroid disorders) to 11.0% for all types of current thyroid disorders and 7.1% or 3.9% for current hyperthyroidism or current hypothyroidism only. Indeed, all of these values surpass that of 1.7% reported in the ISCVT.

The pathophysiological basis for an association between CVST and thyroid disorders (especially hyperthyroidism) has varied explanations that are mainly based on several case reports. Studies on haemostatic changes in patients with thyroid disorders still lack unambiguous results and sometimes are accompanied by methodological shortcomings [for review, see Erem (7) Franchini (9)]. One study attributed the higher thromboembolic potential in hyperthyroid patients to endothelial activation and decreased fibrinolytic activity (6), whereas another described accelerated platelet plug formation via elevated levels of von Willebrand factor as the underlying cause for coagulation abnormalities (26). In addition, increases in the levels of tissue factor as well as thrombin and plasmin activity (6, 27, 28) were described as factors that contribute to hypercoagulability in hyperthyroidism (7).

In contrast, various pathophysiological mechanisms associated with an increased risk of bleeding or hypocoagulability have been found for overt hypothyroidism. These include a decreased platelet count, platelet adhesion and aggregation, reduced levels of fibrinogen, and increases in fibrinolytic and plasminogen activities (29). In our cohort, six out of the seven patients with hypothyroidism had subclinical hypothyroidism at the time of CVST, which is defined as elevated TSH with normal levels of free T4 and T3. The major prevalence of subclinical hypothyroidism in our CVST patients is in line with the reported association between subclinical/mild hypothyroidism and a prothrombotic state. Possible pathophysiological mechanisms for this include increased levels of fibrinogen and factor VII, decreased levels of antithrombin III, and reduced global fibrinolytic capacity and activity (29).

Due to the lack of an unambiguous pathophysiological explanation, it is still unclear whether the actual hypo/hyperthyroidal state contributes to a hypothetical prothrombotic/hypercoagulable state, or whether a potential underlying autoimmune disease also plays a role, irrespective of the actual thyroidal state. Due to the limited availability of relevant information, our analysis could only distinguish between current and previous thyroid disease by means of thyroid parameters available at the timepoint of CVST. Based on this, we found current hyperthyroidism in 7.1% of patients, and current hypothyroidism in 3.9%.

The main limitation of our study is the retrospective approach. Due to the fact that we only used data and documents stored in the local electronic documentation system, we cannot exclude that relevant information or findings in the history or further disease course of the patients were missed. This potential lack of information may have led to an underestimation of the prevalence of all risk factors, as well as thyroid diseases and other conditions. In addition, our approach was purely based on numbers or prevalence, without drawing on pathophysiological explanations. Therefore, we cannot prove a direct cause-and-effect link between thyroid diseases and CVST.

Besides the lack of a direct cause-and-effect-link, more than half (*n* = 22/38) the patients with some form of current or previous thyroid disease had at least one further risk factor. Thus, the role of thyroid disorders as either a risk factor for, or cause of, CVST is debatable, at least in these cases. However, in the patient collective with risk factors other than thyroid disease, a relevant proportion of patients was also found to have more than one risk factor (*n* = 60/144). A large proportion of CVST-patients potentially do not have a single specific risk factor (particularly CVST patients with concomitant thyroid disease), but rather a combination of two or more risk factors that leads to a hypercoagulable state, which, in turn, ultimately results in CVST.

In summary, our results provide numerical evidence for the assumed association between thyroid diseases (particularly hyperthyroidism) and CVST. Our study suggests that this may be of higher relevance (due to higher prevalence) than previously reported. In contrast to several other risk factors for CVST, the majority of thyroid disorders are treatable in a simple and effective manner. Therefore, we suggest considering thyroid dysfunction as a relevant risk factor for CVST, and propose the evaluation of thyroid parameters in patients with CVST by default.

## Data Availability Statement

The raw data supporting the conclusions of this article will be made available by the authors, without undue reservation.

## Ethics Statement

The studies involving human participants were reviewed and approved by Ethics committee of the University of Freiburg, Germany. Written informed consent for participation was not required for this study in accordance with the national legislation and the institutional requirements.

## Author Contributions

MH: study concept, acquisition, analysis, interpreting of data, drafting, and revision of the manuscript. CK: acquisition of data, critical revision and final approval of the manuscript. CW: critical revision and final approval of the manuscript. JL: study concept, interpreting of data, critical revision, and final approval of the manuscript. All authors contributed to the article and approved the submitted version.

## Conflict of Interest

The authors declare that the research was conducted in the absence of any commercial or financial relationships that could be construed as a potential conflict of interest.
